# Scurvy in a 29-Month-Old Patient Presenting With a Gower Sign

**DOI:** 10.7759/cureus.29342

**Published:** 2022-09-19

**Authors:** Carlos A Monroig-Rivera, Keishla C Valentín-Martínez, Edwin Portalatín-Pérez

**Affiliations:** 1 Department of Medicine, Ponce Health Sciences University, Ponce, PRI; 2 Department of Orthopedic Surgery, Mayagüez Medical Center, Mayagüez, PRI

**Keywords:** gower sign, antalgic gait, pediatrics, vitamin c, scurvy

## Abstract

Scurvy is a preventable condition caused by a severe vitamin C deficiency for prolonged periods. Most literature cases describe children with neurobehavioral disorders or extreme dietary restrictions. Vitamin C deficiency may be a rare clinical presentation in the developed world; hence, it is often overlooked and can lead to extensive workups when the history alone could have raised suspicion for the diagnosis. We report a previously healthy 29-month-old boy initially admitted to the hospital due to loss of ambulation over a three-week course. The patient had no history of fever, and the inflammatory parameters were normal. Blood workup, plain radiographs, and magnetic resonance imaging (MRI) of the right lower extremity were unremarkable. The patient was discharged home with antibiotics and anti-inflammatory medication but arrived a week later with worsening lower extremity weakness leading to complete loss of ambulation. Vitamin C deficiency was confirmed to be below normal levels (<0.4mg/dL), and a diagnosis of scurvy was confirmed and treated with oral ascorbic acid. Subsequently, his mother brought him to the orthopedic clinic with a positive Gower sign. CPK levels were normal. Within a month of ascorbic acid replacement, all symptoms disappeared. Our patient was a picky eater, which emphasizes the importance of early dietary screening to discover the underlying cause of symptoms. Vitamin C deficiency should be part of the differential diagnosis in patients with unremarkable laboratory workup for infection and other diseases presenting with a Gower sign.

## Introduction

Vitamin C, also known as ascorbic acid, is a water-soluble vitamin that, unlike most mammals, humans cannot produce on their own [1]. Vitamin C is needed as a cofactor for many biochemical pathways and is essential to maintain the standard structure and function of teeth, bones, and connective tissues [2]. In addition, it supports the immune system by enhancing cellular processes such as phagocytosis and apoptosis. Vitamin C deficiency, also known as scurvy, affects collagen biosynthesis due to inefficient hydroxylation in specific proline and lysine residues necessary for forming collagen triple helix. It has been known since ancient Greek and has existed for more than three million years [3]. It may occur secondary to reduced intake, reduced absorption, or a metabolic condition that prevents in vivo conversion to a functional form [4]. However, this condition has become forgotten in modern society due to the absence of apparent comorbidities and risk factors [3-6]. Most cases described in the literature have been in the aging population, alcoholics, and those with a limited diet consisting primarily of toast and tea [3]. In children, similar restrictions may be less obvious but just as expected, such as neurobehavioral disorders, a selective or restricted diet, and delayed weaning in infancy to solid foods containing vitamin C [7].

Reported symptoms include fatigue, weight loss, bone pain, swelling over long bones, gingival swelling, bleeding of gums, petechial hemorrhage, and corkscrew hair [8-10]. As vitamin C deficiency may be a rare clinical presentation in the modern day, it is common to miss the diagnosis initially. As a result, it leads to unnecessary investigations and excessive clinical workup when the history alone could have raised suspicion. This report focuses on a rare presentation of a Gower sign appearing in a 29-month-old male patient who was previously healthy and had an associated history of progressive lower extremity weakness leading to complete loss of ambulation and a confirmed diagnosis of scurvy.

## Case presentation

We report a 29-month-old boy who presented to an outpatient orthopedic clinic due to a two-month progressive bilateral lower extremity weakness. The boy was born at term with no complications and had been achieving his developmental milestones. There was no history of trauma or falls before developing his symptoms. As per the mother, the patient had developed a significant right leg limp and an anterior tibia swelling two months before the physical encounter at the orthopedic clinic. At first, the mother took him to another orthopedic surgeon, who recommended admission to the hospital for advanced imaging.

**Figure 1:**
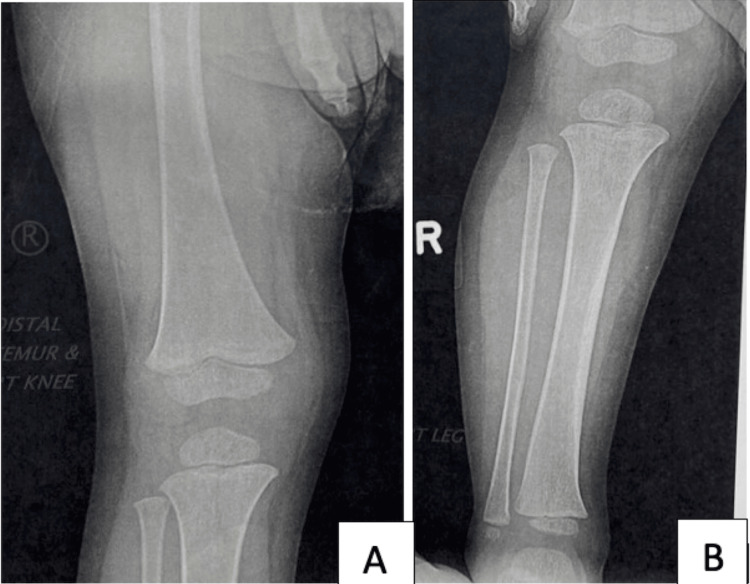
Pediatric patient diagnosed with scurvy. (A, B) Right lower extremity anteroposterior radiograph of a 29-month-old boy was found unremarkable.

Plain radiographs (Figures 1A, 1B) of the right lower extremity were normal. Parallel, an MRI of the lower limbs did not reveal any acute abnormality. The blood workup, which included blood culture, was unremarkable. Regardless, the patient was prescribed anti-inflammatory medication and antibiotics with no impact on the progression of the lower extremity weakness.

The patient was bedridden three weeks after they identified the right tibia swelling. Mother was worried about the clinical scenario and requested an evaluation from various specialists. At the rheumatologist, who diagnosed him with scurvy, the patient presented with gingival bleeding and multiple lower extremity hematomas. The rheumatologist's extensive history mentioned that the patient was a “picky eater,” and his diet consisted mainly of pasta and rice. While pending laboratory results of ascorbic acid, acylcarnitine, pyruvate and lactic acid levels, the rheumatologist decided to treat him empirically with oral vitamin C supplementation. Once confirmed, his serum ascorbic acid level was below standard measures (<0.1mg/dL).

**Figure 2:**
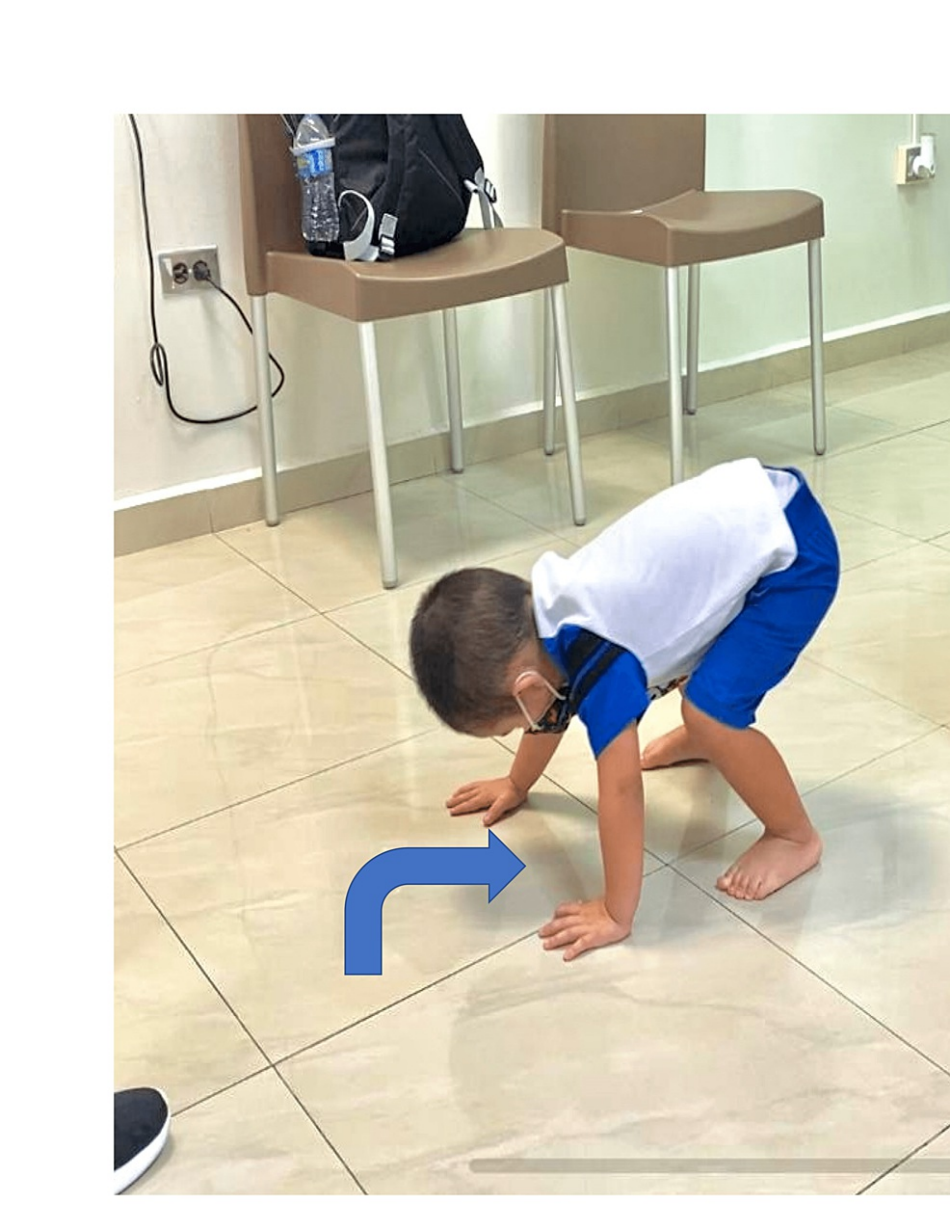
Photograph of the patient showing a positive Gower sign.

Four weeks after the rheumatologist evaluation and the start of oral vitamin C supplementation, the patient continued to have bilateral lower extremity weakness and arrived at the pediatric orthopedic surgeon clinic for evaluation. The exam revealed that both upper and lower extremities had a regular gross neurological examination. There were no signs of active infection, such as localized erythema or tenderness to palpation. The musculoskeletal exam was remarkable for a positive Gower sign (Figure 2). Due to this finding, the orthopedist ordered a creatinine phosphokinase (CPK) level to rule out muscular dystrophy. A summary of the complete biochemical profile of this patient is in Table 1.

**Table 1:** Summary of the biochemical investigations done for this patient.

Investigation	Results (reference range)
Creatinine Phosphokinase (CPK)	119U/L (43-202)
Serum ascorbic acid	<0.1mg/dL (0.4-2.0)
Plasma acylcarnitine profile	Normal
Serum lactic acid	26.4mg/dL (6.3-33.0)
Serum pyruvate	0.5mg/dL (0.3-0.7)

One month later, at the follow-up visit with the orthopedist, the patient's proximal thigh weakness was improving, and no Gower sign was visible on the physical examination. His CPK level returned within normal limits, and he gained considerable weight and improved symptoms since starting vitamin C supplementation. His mother was counseled about the course of the disease and recommended follow-up with his nutritionist to maintain adequate nutritional supplementation to avoid symptoms recurrence. We expect to see him continue improving his symptoms over time. From an orthopedic standpoint, our suggestion included typical activity without any physical restriction.

## Discussion

Scurvy, a severe vitamin C deficiency, is a preventable disease caused by poor intake or poor absorption of vitamin C. Therefore, vitamin C has become an essential exogenous vitamin easily accessible from ascorbic acid-rich food such as fruits and vegetables [1]. Humans lack the gene for L-gluconolactone oxidase (GLO), responsible for the in vivo conversion of ascorbic acid from glucose [1]. Ascorbic acid is pivotal in multiple biochemical pathways to maintain typical bone structure and healthy connective tissue [3]. In humans, vitamin C is a necessary cofactor for prolyl and lysyl hydroxylase, crucial enzymes in collagen biosynthesis [3]. Patients with chronic restriction of vitamin C could present with impaired bone formation and capillary fragility resulting in vascular damage and presenting petechiae, ecchymosis, and gingival bleeding [4]. In addition, patients could present with neurological manifestations such as hyperreflexia, neuralgia, and decreased pinprick sensation [11].

Pediatric patients often suffer from musculoskeletal manifestations [9]. As previously reported, children could present with a progressive antalgic gait, limping, swelling, pain, progressive musculoskeletal weakness, and bedridden behaviors [9]. Hence, muscular weakness and swelling could be ascribed to damage to synovial blood vessels causing bleeding within joints and muscles [9]. With a deficiency of vitamin C for prolonged periods, osteoporosis may develop due to defective osteoid matrix formation and increased bone resorption [9]. Furthermore, literature reports neuropsychiatric symptoms, including irritability and sleep disturbances [8].

A Gower sign is common in hereditary conditions such as Duchenne or Becker muscular dystrophy, not in scurvy. However, it is not clear in the literature whether the Gower sign could be a normal finding in children before three years old. To our best knowledge, this unusual presentation of the Gower sign has been reported in a scurvy patient only once in the scientific literature. Through one anecdotal report, a previously healthy girl was evaluated in an outpatient rheumatology clinic for persistent limping. An extensive workup and plain radiograph of her right lower extremity demonstrated mild osteopenia. She was discharged home on a non-steroidal anti-inflammatory medication (NSAID) with the plan to obtain additional imaging. After seven days, she developed progressive weakness with parental videos showing the presence of a Gower sign and difficulty ascending stairs [1]. Similarly, our pediatric patient presented to the pediatric orthopedic surgeon with a positive Gower sign after weeks of various admissions to the hospital and evaluation with a rheumatologist. Again, we believe this is an unusual manifestation of scurvy in the pediatric population.

Our patient was developing appropriately without any signs of autistic behaviors or neuro-developmental delays placing scurvy on the bottom line of the list of differential diagnoses. Through this case, we highlight the importance of an extensive dietary history in a pediatric patient presenting with a Gower sign. Substantial history-taking skills could lead to important information, such as limited nutritional habits, which could lead to the underlying cause of the symptoms. We are glimpsing an epidemic of overeating and a sedentary lifestyle, which has increased the prevalence of obesity and diabetes in the pediatric population [10]. A small amount of information about inadequate dietary restriction and picky eating behaviors of previously healthy children has been reported [10].

The eating habit of rice and pasta was the root cause that caused our patient to develop scurvy. Scurvy has been known previously as a historical disease [4]. Increased incidence of autism, psychiatric disorders, and severe dietary restrictions seem to introduce back scurvy and vitamin deficiencies to the forefront of pediatric diseases [4]. For patients with progressive musculoskeletal weakness and a Gower sign-on physical exam, we encourage a thorough dietary history and a basic initial workup that includes vitamin C levels. If concerned about poor vitamin C intake or starting to develop musculoskeletal complaints of unknown origin, we recommend giving empiric oral vitamin C while waiting for laboratory results to rule out infectious, inflammatory, or traumatic causes of muscle weakness and swelling. This case emphasizes the requirement of owning scurvy as a possible diagnosis in patients with unexplained progressive muscle weakness and a positive Gower sign. One month later, at the follow-up evaluation, he was working on his diet with a nutritionist and showing clinical improvement in his symptoms.

## Conclusions

This case highlights scurvy as a rare potential cause of a Gower sign in the pediatric population. It is noteworthy because our patient presents without any neuro-developmental disorders and achieving all appropriate milestones. Vitamin C deficiency should be in the differential diagnosis in patients with remarkable laboratory workup for infectious causes and other diseases presenting with an unexplainable Gower sign. We recommend a detailed dietary history in all pediatric patients presenting with nonspecific musculoskeletal complaints and a Gower sign to avoid unnecessary delay in diagnosis.
